# A novel double-stranded RNA mycovirus isolated from *Trichoderma harzianum*

**DOI:** 10.1186/s12985-019-1213-x

**Published:** 2019-09-11

**Authors:** Chenchen Liu, Mei Li, Estifanos Tsegaye Redda, Jie Mei, Jiantai Zhang, Beilei Wu, Xiliang Jiang

**Affiliations:** Institute of Plant Protection, Chinese Academy of Agricultural Sciences, No.2 West Yuanmingyuan Rd., Haidian District, Beijing, 100193 People’s Republic of China

**Keywords:** Mycovirus, *Trichoderma harzianum*, dsRNA, Trichoderma harzianum mycovirus 1

## Abstract

**Background:**

*Trichoderma* spp. are used extensively in agriculture as biological control agents to prevent soil-borne plant diseases. In recent years, mycoviruses from fungi have attracted increasing attention due to their effects on their hosts, but *Trichoderma* mycoviruses have not been the subject of extensive study. We sought to discover novel mycoviruses from *Trichoderma* spp*.* and to determine the effects of the biocontrol function of *Trichoderma* spp.

**Methods:**

Mycoviruses were screened by dsRNA extraction and metagenomic analysis. RT-PCR, 5′ RACE, and 3′ RACE were used to obtain the genome sequence. MEGA software was used to classify the new mycovirus. The effects of the identified mycovirus on the biological properties of the host strain 525 were evaluated using cucumber plants and *Fusarium oxysporum* f. sp. *cucumerinum*.

**Results:**

A novel mycovirus, Trichoderma harzianum mycovirus 1 (ThMV1) (accession number MH155602), was discovered in *Trichoderma harzianum* strain 525, a soil-borne fungus collected from Inner Mongolia, China. The mycovirus exhibited a double-stranded RNA (dsRNA) genome with a complete genome sequence of 3160 base pairs and two open reading frames (ORFs) on the negative strand. Phylogenetic analysis indicated that it belongs to an unclassified family of dsRNA mycoviruses. The removal of ThMV1 from the host 525 strain reduced host biomass production and improved the biocontrol capability of the host for *Fusarium oxysporum* f. sp. *cucumerinum*. At same time, the presence of ThMV1 improved the growth of cucumber.

**Conclusion:**

ThMV1 is a new unclassified mycovirus found in *T. harzianum*. It not only affects the phenotype of the host strain but also reduces its biocontrol function, which sheds light on the interaction between the mycovirus and *Trichoderma* spp.

**Electronic supplementary material:**

The online version of this article (10.1186/s12985-019-1213-x) contains supplementary material, which is available to authorized users.

## Background

Mycoviruses are widespread viruses that infect filamentous fungi and yeasts, and most do not cause their hosts to exhibit obvious symptoms [1, 2]. The majority of mycoviruses exhibit a double-stranded RNA (dsRNA) genome, which is diagnostic. However, a few mycoviruses exhibit single-stranded RNA (ssRNA), double-stranded DNA (dsDNA), or single-stranded DNA (ssDNA) genomes [3]. Based on the mode of replication and the type of genome, the International Committee on Taxonomy of Viruses has divided all currently known mycoviruses into 16 families and an unclassified group. The 16 families include seven dsRNA virus families, five positive-sense ssRNA virus families, two reverse-transcription virus families (+ssRNA), one negative-sense ssRNA virus family, and one positive-sense ssDNA virus family [4]. The taxonomic status of approximately 20% of fungal viruses has yet to be determined [5, 6].

Mycoviruses are transferred from one fungal isolate to another through hyphal fusion (anastomosis) or from parent to offspring via spores (mostly via fusion of asexual spores but also sexual spores). Some mycoviruses can be eliminated in sexual spores. For example, ssRNA and dsRNA viruses in Basidiomycetes can be transmitted by basidiospores, but they can also be removed from the basidiospores produced by *Helicobasidium mompa* [7]. In some cases, they can be virulent to their hosts, but most mycovirus infections are asymptomatic [8].

In recent years, mycoviruses have drawn increased attention for the effects they can have on their host virulence [9]; some can significantly reduce virulence (e.g., the dsRNA virus DK21 of *Fusarium graminearum* [10]), while others can significantly increase it (e.g., the dsRNA mycovirus RsRV2 found in the rice sheath blight isolate D122 strain [11]). These findings have spurred the development of hypovirulence-associated mycoviruses for use as biocontrol agents [12–14].

Mycoviruses exhibit two main mechanisms that facilitate their function as biocontrol agents of plant pathogenic fungi: first, they can cause the host to become a low-virulence strain; second, the metabolites induced by the mycovirus can increase the pathogenicity of the host [15–17]. Although some low-virulence mycoviruses have been found in phytopathogenic fungi, most are still in the research stage examining their potential as biocontrol agents. The most successful mycovirus biocontrol agent identified to date has been *Cryphonectria parasitica* hypovirus 1 (CHV1), which was the first low-virulence mycovirus employed to both prevent and treat disease [18].

*Trichoderma* spp. are anamorphic soil-borne filamentous fungi classified as belonging to phylum *Ascomycota*, class *Sordariomycetes*, order *Hypocreales*, and family *Hypocreales.* While they are well studied [19], their mycoviruses have not been sufficiently investigated [20].

Here, we chose 150 strains of *Trichoderma* spp. isolated from Xinjiang, Inner Mongolia, Jilin, and Heilongjiang Provinces, China, to screen mycoviruses using metagenomics and molecular biology methods. One mycovirus was recovered, and its genome and molecular characteristics are described along with its biological properties.

## Methods

### Fungal strains

One hundred and fifty *Trichoderma* spp. strains were isolated from the forests and grasslands of Xinjiang, Inner Mongolia, Jilin, and Heilongjiang Provinces, China, in 2014–2016 (Additional file 1: Table S1). All fungal strains were cultured on potato dextrose agar (PDA) plates for 7 days at 28 °C.

### Extraction and purification of dsRNA

dsRNA was extracted from the mycelia using CF-11 cellulose column chromatography [21]. To isolate dsRNA, the mycelia were grown for 7–10 days on cellophane membranes placed on top of the PDA medium in Petri dishes. Following harvest, the mycelia were stored at − 80 °C. dsRNA was extracted according to a previously described method [21] and then treated with RNase-free DNase Ι (TaKaRa, Dalian, China) and S1 Nuclease (TaKaRa, Dalian, China) following the manufacturer’s instructions to remove any DNA and ssRNA contamination. Finally, electrophoresis in a 1.5% agarose gel was performed, and t the dsRNA was detected by UV transillumination.

### Metagenomic sequencing

dsRNA was extracted and sequenced using high-throughput sequencing (Illumina HiSeq 2000/2500) at the Biotechnology Corporation (Biotechnology Corporation, Shanghai, China). Briefly, purified dsRNA was first assessed for quality. Ribosomal RNA (rRNA) was removed, and the dsRNA was fragmented. Next, first-strand complementary DNAs (cDNAs) were synthesized, followed by second-strand cDNAs. The complete sample sequencing library was obtained, and the full library was sequenced. The sequencing data were then analysed; unigenes were annotated with RefSeq; and non-redundant proteins according to the National Center for Biotechnology Information (NCBI) Basic Local Alignment Search Tool (BLAST) were searched to screen mycovirus-related sequences.

### Cloning and sequencing of the mycovirus

cDNA of the dsRNA were obtained by reverse-transcription polymerase chain reaction (RT-PCR) based on the contig obtained from metagenomics analysis (forward primer: 5′ TCGATGTACGGATTCTCGTGC 3′; reverse primer: 5′ TGTCATCATCGTCTTCAGCCC 3′; fragment length: 2997 bp). Having obtained most of the virus sequence, the 5′- and the 3′-termini were cloned using the rapid amplification of cDNA ends (RACE) method. The 5′-end was obtained by the classic 5′-RACE cloning method [22] (Additional file 1: Table S3). A poly(A) tail was added to the 3′-end of the cDNA using terminal deoxynucleotidyl transferase (TDT). Linker primers and specific primers were used to perform nested PCR amplification. The 3′-end of the virus was obtained through the 3′-RACE cloning method [23] (Additional file 1: Table S3). Using the QT hybrid primer software, specific primers were designed for nested PCR amplification.

The RT-PCR products were purified using a gel extraction kit (Tiangen, Germany), ligated to the PMD18-T vector, and submitted for sequencing. Using DNAMAN software, the cDNA clone sequence of the virus and the RACE clone sequence were assembled to form a complete viral genome sequence.

### Complete genome sequence and phylogenetic analysis

Mycovirus ORFs were identified by the ORF finder program from the NCBI website (https://www.ncbi.nlm.nih.gov/orffinder/). The mycovirus sequence was searched using the NCBI BLAST program to identify similar mycoviruses for phylogenetic analysis. The phylogenetic tree was constructed using the maximum likelihood method for the 29 chosen mycovirus sequences of RdRP, 22 mycovirus sequences of hypothetical proteins or CPs, and 22 RdRP+ hypothetical proteins or CPs using MEGA 6.0 software based on the best models of nucleotide substitution: K2 + G + I, LG + G + I + F, and rtREV+G + I + F (Additional file 1: Tables S4, S5 and S6).

### Effect of the mycovirus on the biological properties of *Trichoderma* strain 525

Tthe inhibitory function of ribavirin was used to establish an analysis system for eliminating mycovirus from *Trichoderma* strain 525 by protoplasting/regeneration. The enzymatic hydrolysate used for the preparation of protoplasts of *Trichoderma* strain 525 was *Trichoderma* lysing enzyme (BingDa Biotechnology Company, Beijing). After enzymatic lysis, a 200 μL aliquot of protoplasts was applied to regeneration medium that contained ribavirin (100 μM) (BingDa Biotechnology Company, Beijing), followed by culture at 28 °C for 2 days. Single colonies were then picked and transferred to PDA medium that contained ribavirin at 100 μM, followed by culture for 7 days. To check if the mycovirus was present, dsRNA was extracted [24], after which RT-PCR and northern blotting were used to reconfirm whether dsRNA had been eliminated from the *Trichoderma* strain. A digoxigenin (DIG)-labelled probe was synthetized using the PCR DIG Probe Synthesis Kit (Roche Diagnostics, USA) with the following RT-PCR primers: forward primer: 5′ TCGATGTACGGATTCTCGTGC 3′; reverse primer: 5′ TGTCATCATCGTCTTCAGCCC 3′. The length of the RT-PCR fragment was 2997 bp.

The biological functions of the 525 strain with the mycovirus (T525) and the strain without the mycovirus (T525-F) were evaluated. Both strains were cultured in PDA, corn meal dextrose agar (CMD) medium, and Czapek–Dox (CZA) medium at 28 °C for 8 days. The length of hyphal growth was measured every 24 h, with thirty replicates in each case. To obtain the differences between the strains, the hyphal growth rates of the two strains were compared with each other. The T525 and T525-F strains were inoculated into PD liquid medium and incubated at 28 °C with 180 rpm shaking for 2 days. The dry weights of the mycelia in each bottle were then determined, and the biomasses of the two strains were compared, with twenty replicates in each case.

To evaluate biocontrol capabilities in vitro, antagonistic experiments involving T525 and T525-F were carried out. The three pathogens *Fusarium oxysporum* f.sp. *cucumebrium* Owen, *Botrytis cinerea,* and *Fusarium oxysporum* f. sp. *vasinfectum* were chosen for dual culture to analyse the characteristics of the antagonism of T525 and T525-F.

The biocontrol effect of T525 and T525-F against *F. oxysporum* f. sp. *cucumerinum* was further explored in vivo in cucumber plants. Six treatments were designed (Additional file 1: Figure S4): T1, in which cucumber seeds were planted in plates with sterilized water and incubated at 28 °C until the 6th day, after which the seedlings were transferred to glass test tubes (GTTs) containing 10 mL of 1/8 MS; T2, in which cucumber seeds were planted in plates with sterilized water and then incubated at 28 °C until the 4th day, after which the seedlings were exposed with 1 × 10^7^ CFU/mL spores of *F. oxysporum* f. sp. *cucumerinum* by dipping, cultured for one day, and then transferred to GTTs containing 10 mL of 1/8 MS on the 6th day; T3, in which the cucumber seeds were only treated by dipping with 1 × 10^7^ CFU/mL spores of T525, then transferred to plates with sterilized water and incubated at 28 °C until the 6th day, after which they were transferred to GTTs containing 10 mL of 1/8 MS; T4, in which the cucumber seeds were only treated by dipping with 1 × 10^7^ CFU/mL spores of T525-F, then transferred to plates with sterilized water and incubated at 28 °C until the 6th day, after which they were transferred to GTTs containing 10 mL of 1/8 MS; T5, in which the cucumber seeds were treated by dipping with 1 × 10^7^ CFU/mL spores of T525, then transferred to plates with sterilized water and incubated at 28 °C. On the 4th day, the germinated seedlings were treated by dipping with 1 × 10^7^ CFU/mL spores of *F. oxysporum* f. sp. *cucumerinum* and cultured for one day until the 6th day, when they were transferred to GTTs containing 10 mL of 1/8 MS; T6, in which the cucumber seeds were treated by dipping with 1 × 10^7^ CFU/mL spores of T525, then transferred to plates with sterilized water and incubated at 28 °C. On the 4th day, the germinated seedlings were treated by dipping with 1 × 10^7^ CFU/mL spores of *F. oxysporum* f. sp. *cucumerinum* and cultured for one day, then transferred to GTTs containing 10 mL of 1/8 MS on the 6th day. On the 12th day, all treatments were observed. Every treatment was carried out in three repeat experiments with thirty replicates in total. For treatments T1, T3, and T4, plant growth continued to be observed until the 24th day. The plates for all treatments were filled every day with sterilized water, and GTTs were filled to a constant volume of 10 mL. Every five plants were photographed for every treatment.

## Results

### Sequencing of the mycovirus genome

**Fig. 1:**
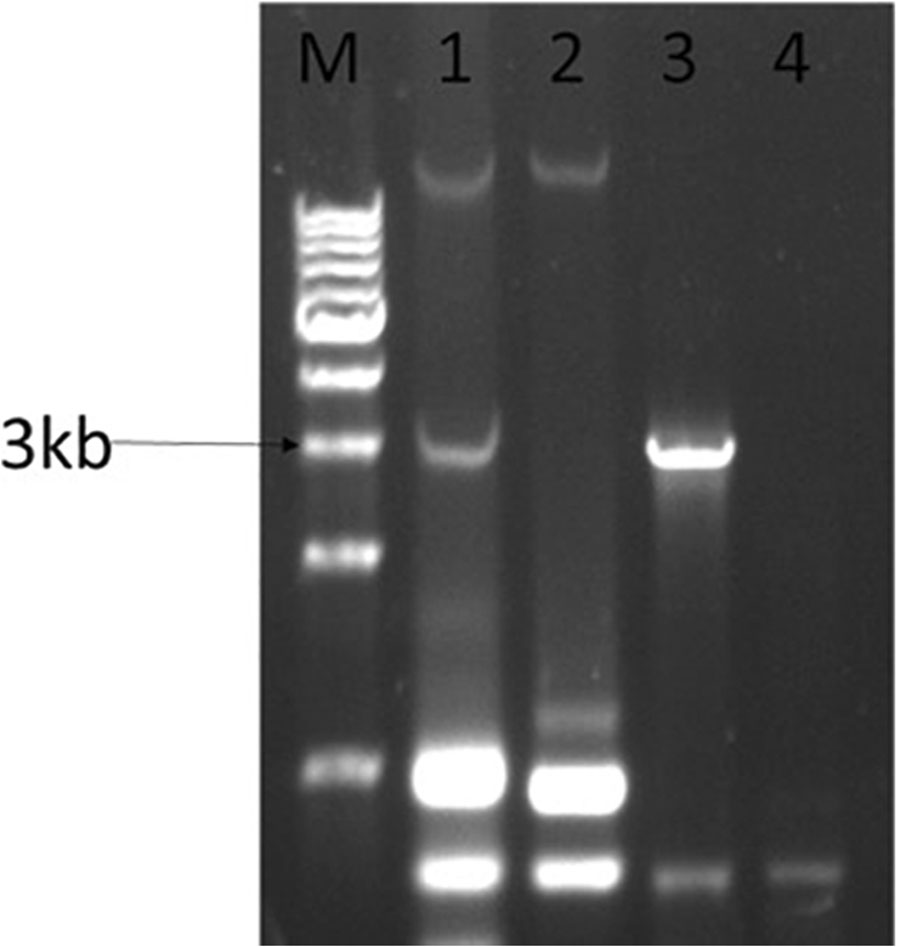
Electrophoresis results. Lane M: DNA marker (10 kb ladder, TaKaRa); Line 1: dsRNA (approximately 3.2 kb) extracted and purified from *T. harzianum* strain 525, subjected to electrophoresis in a 0.8% agarose gel and detected under a UV transilluminator. Lane 2: dsRNA from *T. harzianum* strain 525 after degradation by the enzymes of RNase I and DNase I and electrophoresis in a 0.8% agarose gel, detected under a UV transilluminator. Lane 3: Detection of fragments of the dsRNA segments of mycovirus genomes by RT-PCR using specific primers

From the 150 *Trichoderma* strains, we identified a strain designated HB40525 (or 525), containing typical dsRNAs of mycoviruses. Electrophoresis showed that the putative mycovirus fragment was approximately 3 kb in length (Fig. 1). Using next-generation sequencing (NGS) analysis, the contig-36 was determined to have a size of approximately 3 kb and to show high similarity to three mycoviruses: Alternaria longipes dsRNA virus 1 [25], Penicillium janczewskii Beauveria bassiana-like virus 1 [26], and Beauveria bassiana RNA virus 1 [27]. The strains exhibited sequence homology of 63.50% to Alternaria longipes dsRNA virus 1, YP_009052469.1 [25], 57.30% to Penicillium janczewskii Beauveria bassiana-like virus 1, ALO50135.1 [26] and 57% to Beauveria bassiana RNA virus 1, AKC57301.1 [27] (Additional file 1: Table S2). Using the sequence of contig-36, primers for RT-PCR were designed, and RT-PCR was performed. The cDNA sequences of the mycovirus without the 5′ and 3′ termini were verified. The 5′ and 3′ terminal sequences of the cDNAs were obtained by the classic 5′ RACE and 3′ RACE cloning methods (Fig. 1, Additional file 1: Table S3 and Figure S1). The results indicated that the 3′ terminus of the virus contained 15 poly(A) structures. Additional structures within the 5′ and 3′ termini were evaluated using RNA structure 3.0 (Additional file 1: Figure S2). The cDNA sequences and RACE sequences were assembled to obtain the complete sequence of the virus using DNAMAN software. The resulting mycovirus genome was 3160 bp (including the poly(A) tail) and was submitted to GenBank under accession number MH155602.

### Structure of the mycovirus genome

**Fig. 2:**
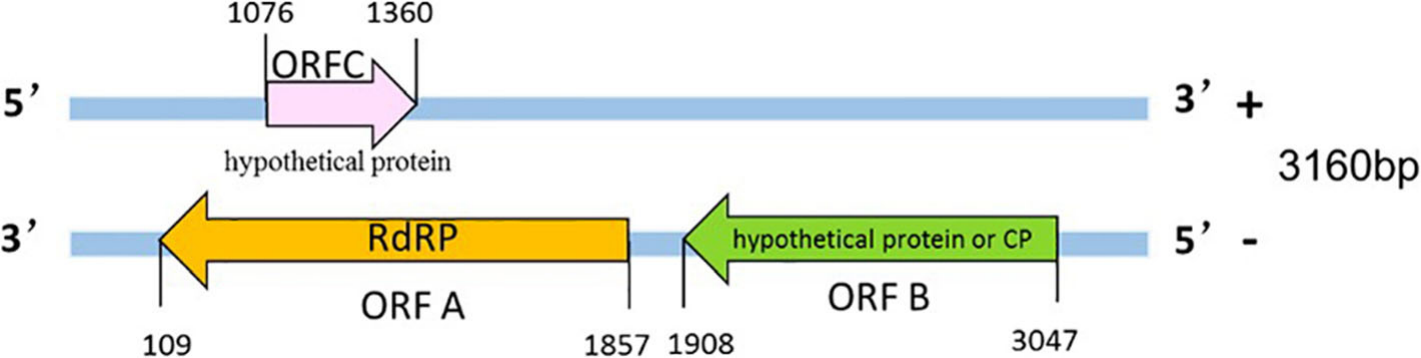
Schematic representation of the genomic organization of ThMV1–525. The THRV1–525 genome is 3160 bp in length and contains two ORFs (ORF-A and ORF-B). ORF-A encodes the RNA-dependent RNA polymerase (RdRp); ORF-B encodes a hypothetical protein or coat protein (CP); the black lines indicate the 5′-UTR, 3′-UTR, and internal region

In the genome sequence, the G + C content was 51.98%. The coding strand of the dsRNA exhibited two ORFs on the negative strand, which constituted the coding strand of the mycovirus. ORF-A (residues 1857–109) encoded an RNA-dependent RNA polymerase (RdRP), which was a protein of 582 amino acids with a molecular weight of approximately 66 kDa. ORF-B (residues 3047–1908) encoded a putative protein of 379 amino acids with a molecular weight of approximately 41 kDa. The positive strand harboured ORF C (residues 1076–1370), presumed to encode a hypothetical protein containing 94 amino acids with a poly(A) structure at the 3′ terminus (Fig. 2, Additional file 1: Figure S2).

### Phylogenetic analysis of the mycovirus and its taxonomic status

**Fig. 3:**
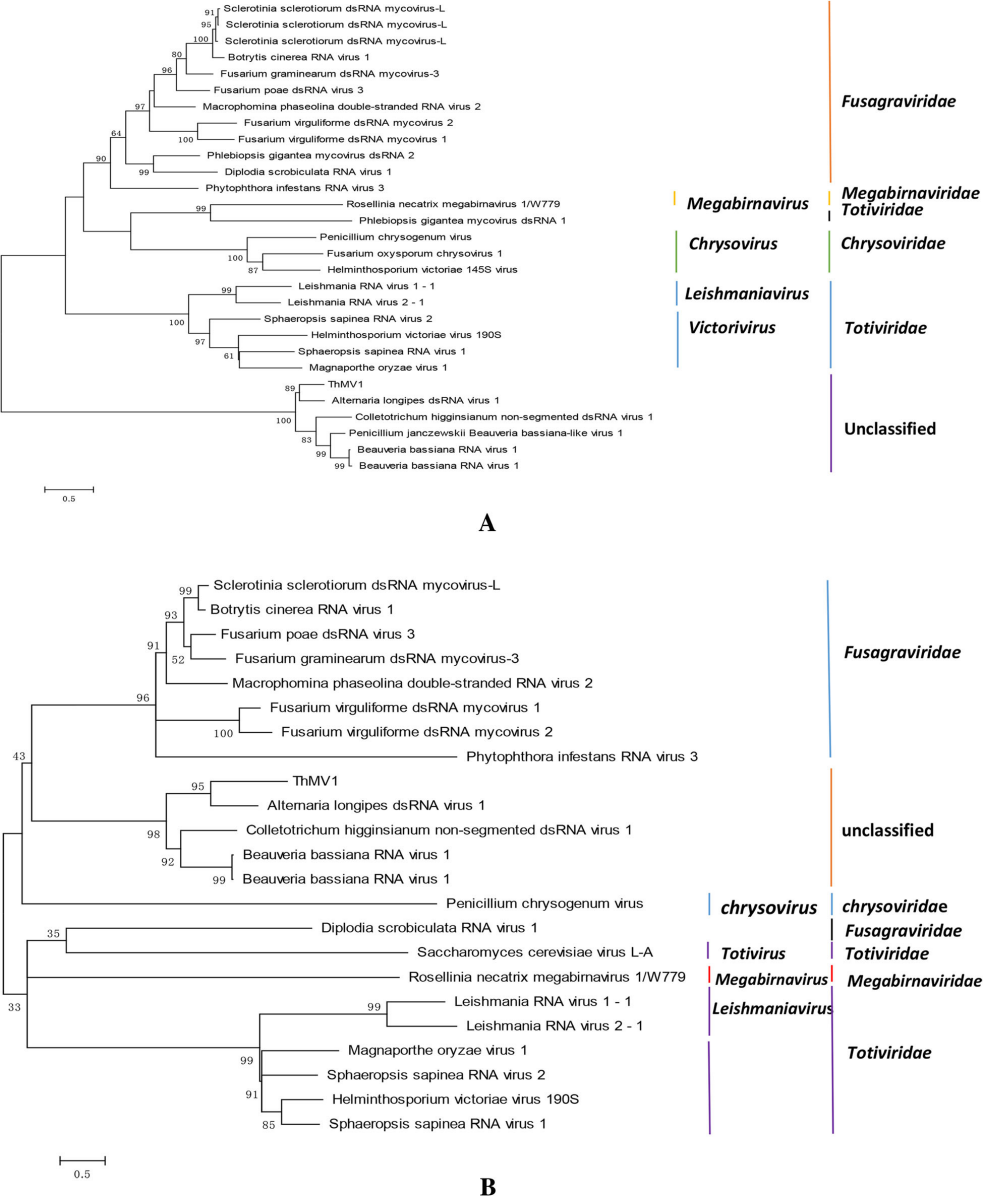
The phylogenetic trees of ORF-A and B constructed by the maximum likelihood method (1000 bootstrap replicates) using the deduced amino acid sequences of the RDRP, hypothetic protein or CP, and RDRP+ hypothetic protein or CP. **a** The phylogenetic tree of ORF-A; **b** the phylogenetic tree of ORF-B

An NCBI BLASTN search identified five closely related fungal viruses with amino acid sequences similar to that of ORF-A: Alternaria longipes dsRNA virus 1 [25] (AlRV1, YP_009052469.1, homologous at 63% sequence identity), Penicillium janczewskii Beauveria bassiana-like virus 1 [26] (PjBlV1, ALO50135.1, homologous at 57% sequence identity); Beauveria bassiana RNA virus 1 [28] (BbNV-1, YP_009154711.1 homologous at 57% sequence identity); Beauveria bassiana RNA virus 1 [27] (BbV1-A24, AKC57301.1, homologous at 57% sequence identity); and Colletotrichum higginsianum non-segmented dsRNA virus 1 [29] (ChNRV1, YP_009177217.1, homologous at 55% sequence identity). The phylogenetic tree with the best model indicated that these fungal mycoviruses belonged to different families (*Fusagraviridae*, *Megabirnaviridae*, *Totiviridae*, *Chrysoviridae*), and the mycovirus of strain 525 belonged to the unclassified group structure [30] (Fig. 3a and Additional file 1: Table S4).

Based on the amino acid sequence of ORF-B, we found five closely related fungal viruses using NCBI BLASTn: AlRV1 (YP_009052468.1 homologous at 41% sequence identity); BbNV-1 (YP_009154710.1 homologous at 42% sequence identity); BbV1-A24 (AKC57300.1, homologous at 44% sequence identity); PjBlV1 (ALO50134.1, homologous at 44% sequence identity); and ChNRV1 (YP_009177216.1, homologous at 43% sequence identity). When all of these sequences were analysed using the best model, LG + G + I + F, ORF-B was grouped with the five mycoviruses with high similarity. The BbNV-1 protein is a putative coat protein, so ORF-B of strain 525 was considered to also encode a coat protein (Fig. 3b and Additional file 1: Table S5).

From the BLAST results for ORF-C based on the amino acid sequences from the NCBI database, only one hypothetical protein from Mycolicibacterium conceptionense (WP_076212170.1) was discovered, with 34% identity.

For the sake of completeness, the alignment that resulted from concatenating the aligned RdRP and coat protein sequences was also used to construct a single maximum likelihood phylogenetic tree (using the rtREV+G + I + F model) (Fig. 3c and Additional file 1: Table S6). The results were identical to the phylogenetic trees inferred using each protein independently. We concluded that the mycovirus from *T. harzianum* strain 525 was novel and unclassified, and we named it Trichoderma harzianum mycovirus 1 (ThMV1).

### Biological effects of ThMV1 on *Trichoderma harzianum* 525

**Fig. 4:**
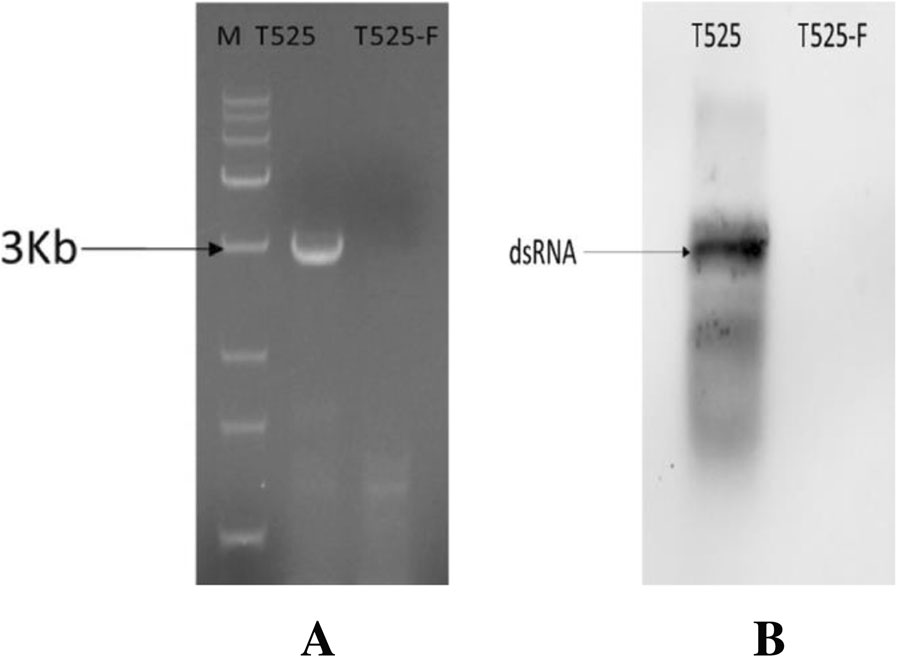
Detection of T525- F by RT-PCR and Northern blotting. **a** Detection of T525 and strain T525-F (virus-free) by RT-PCR using specific primer pairs. **b** Northern blot detection of the dsRNAs of T525 and T525-F (virus-free) using a DIG probe

The elimination of the mycovirus from *Trichoderma* strain 525 was successfully performed using ribavirin and protoplasting/regeneration. RT-PCR of T525 and T525-F was performed to verify the elimination of ThMV1. The results showed that T525 exhibited a fragment length of 2997 bp, but T525-F did not contain the same fragment (Figs. 1 and 4a). No dsRNA from T525-F was detected by Northern blot analysis using a DIG-labelled probe, confirming that the mycovirus was successfully removed from T525-F (Fig. 4b).

**Fig. 5:**
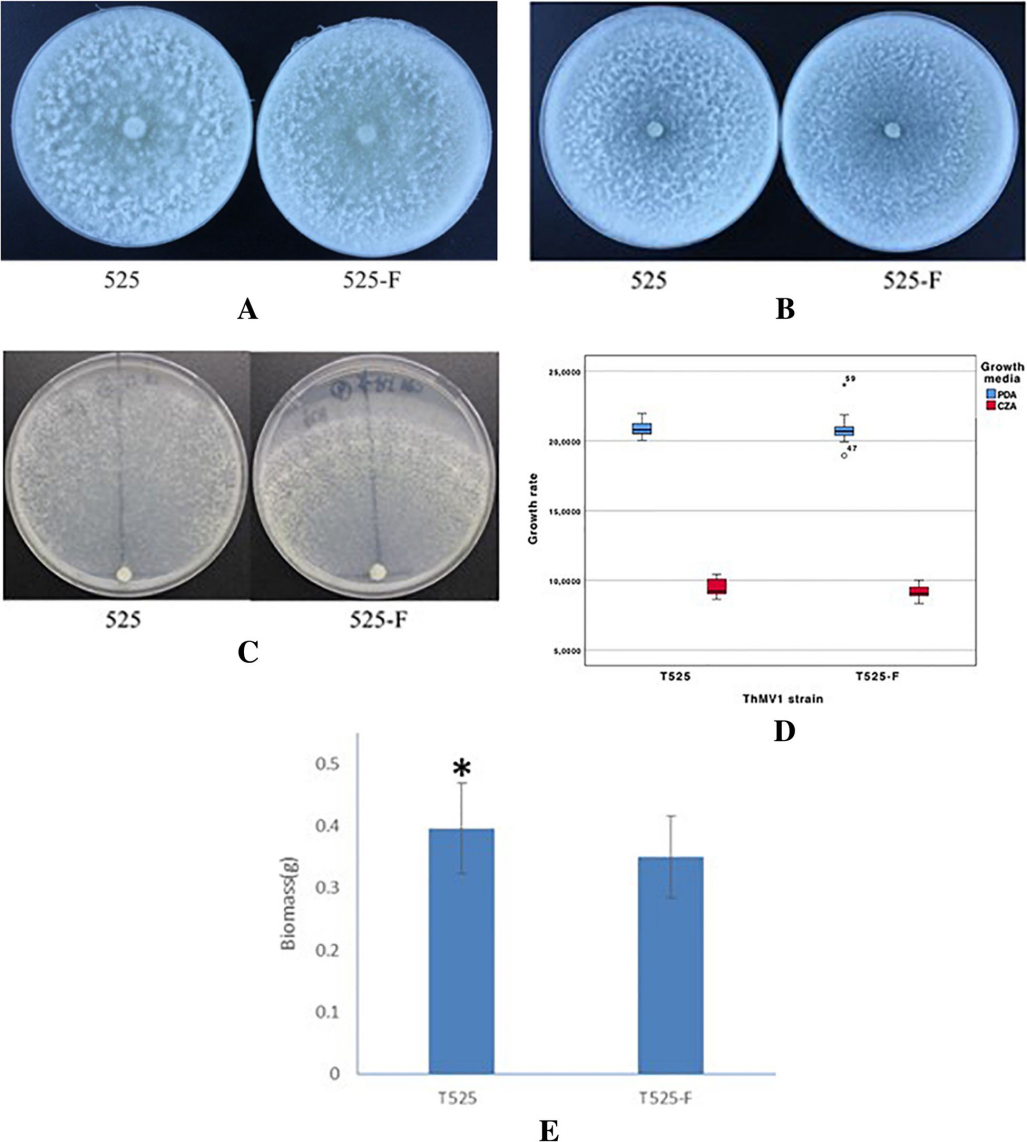
Colony morphology of T525 and T525-F after 7 days of culture on PDA (potato dextrose agar medium), CMD (corn meal dextrose agar medium), and CZA (Czapek–Dox agar medium). **a** PDA medium. **b** CMD medium. **c** CZA medium. **d** Growth rate of strain 525 and strain 525-F after 3 days on PDA and CZA medium, respectively. E: Biomass comparison of T525 and T525-F. The bars (D-F) represent the standard deviation from the mean (**d**: *n* = 30; **e**: *n* = 20). “*” indicates that differences are statistically significant (*p* < 0.05)

When the growth rate of the two strains cultured on different media was measured, we found no statistically significant differences between them (Fig. 5d and Additional file 1: Tables S7, S8, S9, and S10, ANOVA *P* = 0.095), although on the 8th day, the hyphae of T525 had already covered the entire plate, while the hyphae of the T525-F had not yet done so (Fig. 5c). There were differences between the two growth media, however, with the hyphae growing almost twice as fast on PDA as on CZA (Fig. 5a and b and Additional file 1: Tables S7, S8, S9, and S10, ANOVA *P* < 0.001). No significant strain-by-media interaction was observed (Fig. 5d and Additional file 1: Table S9, ANOVA *P* = 0.389), confirming that the similarity of growth rates between the two strains was independent of the medium. Because the CMD medium was thick and opaque, we could not measure the length of the hyphae accurately, but T525 grew more vigorously and densely than T525-F (Fig. 5b).

In contrast to the lack of difference between T525 and T525-F in terms of growth rates, the biomass produced by T525 was 13.25% greater than that of T525-F (Fig. 5e), a difference that was statistically significant (two-sample *t*-test: *t* = 2.117, 38 d.f., *P* = 0.041) (Additional file 1: Tables S8 and S9). Thus, the presence of the mycovirus had a beneficial effect on the production of mycelia.

**Fig. 6:**
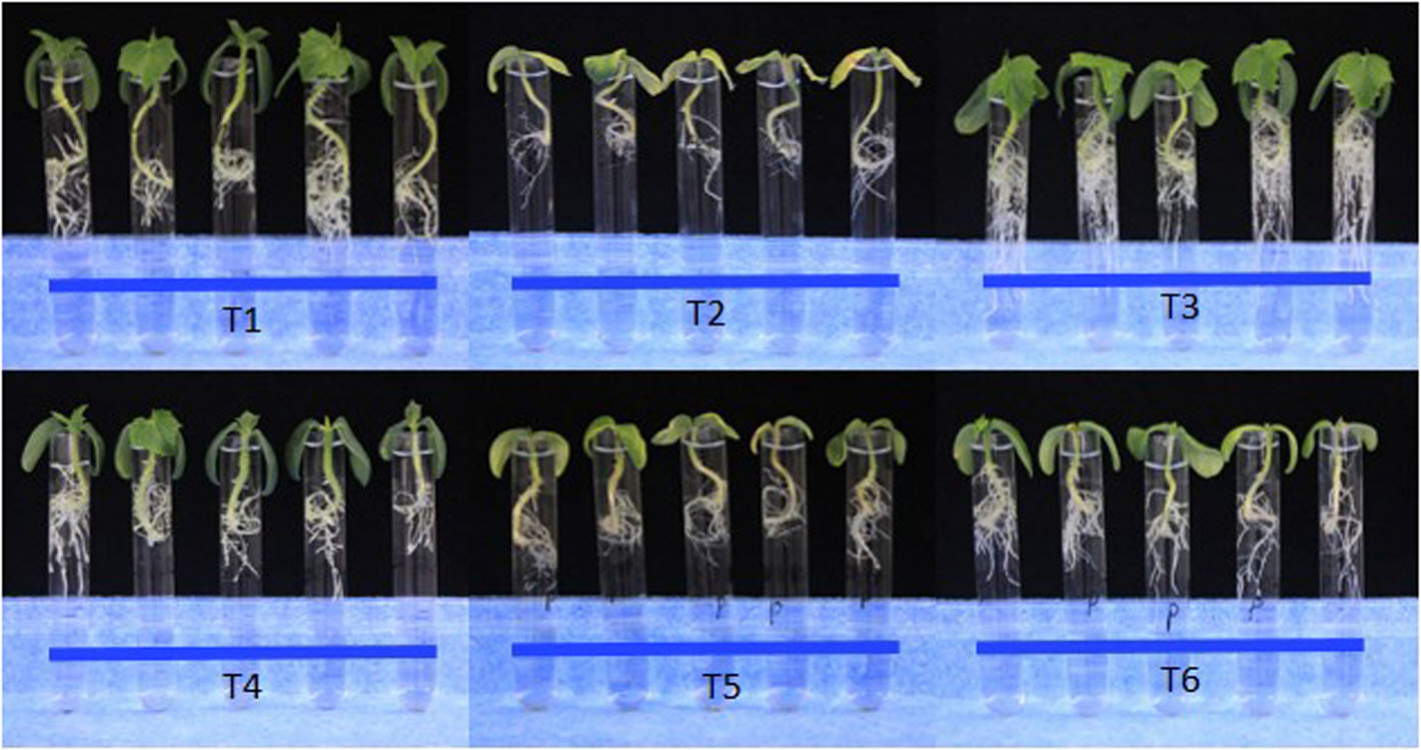
Evaluation of the biocontrol effects of T525 and T525-F. T1: healthy cucumber without any treatment; T2: cucumber seeds treated only with *F. oxysporum* f. sp. *Cucumerinum*; T3: cucumber seeds treated only with *Trichoderma* strain 525; T4: cucumber seeds treated only with *Trichoderma* strain 525-F. T5: cucumber plants treated only with *Trichoderma* strain 525 and *F. oxysporum* f. sp. *Cucumerinum*; T6: cucumber plants treated only with *Trichoderma* strain 525-F and *F. oxysporum* f. sp. *Cucumerinum*

**Fig. 7:**
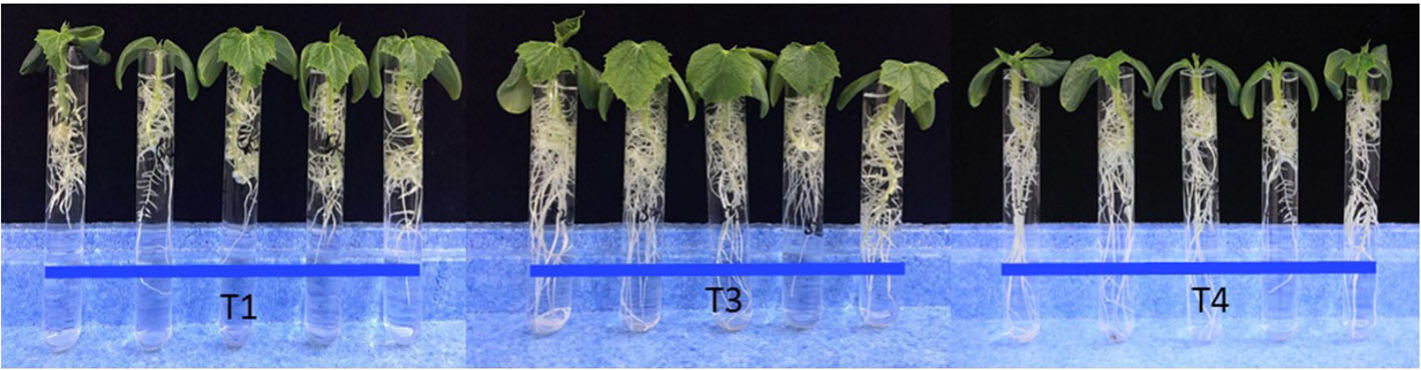
Evaluation of the difference in the improvement of cucumber growth by T525 and T525-F. T1: healthy cucumber without any treatment after 24 d; T3: cucumber seeds treated only with *Trichoderma* strain 525 after 24 d; T4: cucumber seeds treated only with *Trichoderma* strain 525-F after 24 d

To evaluate biocontrol capabilities, we tested the antagonism of T525 and T525-F against three pathogenic fungi (*F. oxysporum* f.sp. *cucumebrium* Owen, *B. cinerea* and *F. oxysporum* f. sp. *vasinfectum*). There were no obvious differences in vitro *(*Additional file 1: Figure S2), but the control effectiveness towards cucumber wilt disease caused by *F. oxysporum* f.sp. *cucumebrium* Owen was significantly different in vivo. While the cucumber seedlings in the T1, T3, and T4 treatments all grew well, little difference existed in the observed growth trend; all of the cucumber seedlings of T3 grew more vigorously and exhibited more fibrous roots than T1 and grew higher than T4 by the 12th day (Fig. 6). Moreover, on the 24th day, the cucumber seedlings of T3 were all larger than those of T1 and T4, with larger true leaves and more fibrous roots (Fig. 7), which indicated that T525 had the potential to function to improve cucumber growth. The T2 seedlings infected only with *F. oxysporum* f. sp. *cucumebrium* Owen were all withered 7 days after inoculation, which indicated that the cucumber species exhibited no resistance to this pathogen. However, the growth trends of T6 were all better than those of T2 and T5. T2 showed severe disease symptoms, whereas the growth trend of T5 only showed the initial stage of withering, with all leaves beginning to turn yellow (Fig. 6 and Additional file 1: Figure S3).

These results together indicate that T525 possesses the capacity to promote plant growth, while T525-F could improve the pathogen resistance of plants. The milder disease symptoms of cucumber seedlings treated with T525-F implied that ThMV1 interacted with its host, T525, and decreased the host’s biocontrol efficiency against plant disease. Plants treated with T525 (T3) appeared to grow significantly better than those that did not receive this treatment (T1) or were treated with T525-F (T4), and T1 was observed to promote better growth than T4.

## Discussion

This study describes a new mycovirus found in *T. harzianum* strain 525, which was isolated from grassland soils in Inner Mongolia, China. Through sequence alignment, the mycovirus was found to exhibit high sequence homology with Alternaria longipes dsRNA virus 1, Penicillium janczewskii Beauveria bassiana-like virus 1, Beauveria bassiana RNA virus 1, Beauveria bassiana RNA virus 1, and Colletotrichum higginsianum non-segmented dsRNA virus 1, with sequence identities of 44–50.5% (Additional file 1: Table S6). Based on phylogenetic analysis of its amino acid sequence, this mycovirus was identified as a new unclassified type of mycovirus.

To date, only three isolates of mycoviruses have been recovered from *Trichoderma* spp. Jom-in and Akarapisan (2009) isolated two mycoviruses with sizes of 0.7 kb and 1.1 kb from *Trichoderma* spp., although their taxonomic status was not analysed or classified [31]. Yun et al. (2016) noted that mycoviruses of *Lentinula edodes* are widespread in fungi [20]. They isolated 32 strains containing dsRNA from 315 Trichoderma strains, which were divided into 15 groups according to dsRNA electrophoresis patterns. Although colony morphological characteristics differed among the groups [20], accurate mycovirus sequences were not obtained. Lee et al. (2017) also isolated an unclassified mycovirus from *T. atroviride* referred to as Trichoderma atroviride mycovirus 1 (TaMV1) [30].

Previous studies on the mycoviruses of fungal pathogens have shown that they can affect the virulence of host fungi. For instance, Li et al. (2015) found that *Fusarium graminearum* Hypovirus 2 (FgHV2/JS16) could significantly reduce the pathogenicity of *Fusarium graminearum* by inhibiting the production of deoxynivalenol (DON) toxin [32]. Zhang et al. (2014) found that the dsRNA mycovirus Rhizoctonia solani RNA virus 2 (RsRV2) from the rice sheath blight isolate D122 strain, which belongs to the family *Paritiviridae,* increased the virulence of host fungus towards plants [11]. Jom-in et al. (2009) tried to remove the mycovirus from TM10 and TM20 hosts but was unable to obtain Trichoderma strain-free mycovirus [31]. Jom-in investigated the antagonistic activity of *Trichoderma* spp. without mycoviruses and indirectly verified that TM10 and TM20 mycoviruses decreased the antagonistic action of *Trichoderma* spp. [31]. In our study, the mycovirus of T525 was successfully eliminated, and ThMV1 did not affect the antagonistic activities of the host in vitro, although it obviously decreased the biocontrol efficiency of T525 against *F. oxysporum* f.sp*. cucumebrium* Owen in vivo. ThMV1 also exhibited the potential to induce plant growth. This study is the first to report the biocontrol traits of a mycovirus from *Trichoderma* spp., highlighting the interactions between *T. harzianum, F. oxysporum* f. sp*. cucumebrium* Owen and ThMV1.

## Conclusions

ThMV1 is the first reported mycovirus that not only affects the biomass of the host strain but also reduces the biocontrol function of *Trichoderma* and promotes the growth of plants. This work provides insight into how to locate and isolate mycoviruses that could potentially affect the biocontrol function of *Trichoderma*. The interaction mechanisms among the mycovirus, *Trichoderma,* pathogen, and plant will be the focus of future investigations.

## Additional file

Additional file 1: **Table S1.** Information on *Trichoderma* strains collected from Xinjiang, Inner Mongolia, Jilin and Heilongjiang Provinces of China. **Table S2.** High identities between contig 36 and other mycoviruses as determined by next-generation sequencing (HGS). **Table S3.** The primers used for 5’ RACE and 3’ RACE of the mycovirus genome sequence. **Table S4.** RdRP data of mycoviruses used in the phylogenetic analysis, including the identities of RdRP between ThMV1 and the compared mycoviruses. **Table S5.** CP data of mycoviruses used in the phylogentic analysis, including the CP identities between ThMV1 and the compared mycoviruses. **Table S6.** RdRP+CP data of mycoviruses used in the phylogenetic analysis, including the identities of RdRP+ CP between ThMV1 and the compared mycoviruses. **Table S7.** Statistical analysis of hyphae from T525 and T525-F after 5 days on PDA medium by the Mann-Whitney U test. **Table S8.** Average growth rate, standard deviation, and statistical analysis of hyphae from T525 and T525-F after 8 days on CZA medium by the Mann-Whitney U test. **Table S9.** Biomass comparison, standard deviation, and statistical analysis of biomass between T525 and T525-F by the Mann-Whitney U test. **Table S10.** Statistical analysis for the comparison of biomass between T525 and T525-F by using SPSS. **Figure S1.** Electrophoresis images of 5’ RACE and 3’ RACE results for the genome sequence of the T525 mycovirus. **Figure S2.** The secondary RNA structures of the 5’UTR and 3’UTR. **Figure S3.** The antagonistic characteristics of T525 and T525-F against *F. oxysporum* f.sp. *cucumebrium* Owen, *B. cinerea* and *F. oxysporum* f. sp. *vasinfectum*. **Figure S4.** Experimental flow chart for evaluating the biocontrol capabilities of *F. oxysporum f. sp. cucumebrium* Owen in cucumber. (DOCX 293 kb)

## Abbreviations

%: Percentage
°C: Degrees Celsius
ANOVA: Analysis of variance
BLAST: Basic Local Alignment Search Tool
bp: Base pair
cDNA: Complementary DNA
CK: Control check
CMD: Corn Meal Dextrose Agar
CP: Coat protein
CZA: Czapek-Dox
DIG: Digoxigenin
dsDNA: Double-strand DNA
dsRNA: Double-stranded RNA
et al.: And others
Fig: Figure
GTT: Glass test tube
i.e.: That is
KB: Kilobase
MEGA: Molecular evolutionary genetics analysis
ml: Millilitre
mm: Millimetre
MS: Murashige and Skoog
NCBI: National Center for Biotechnology Information
NGS: Next-generation sequencing
NO.: Number
ORF: Open reading frame
P: *P* value
PCR: Polymerase chain reaction
PDA: Potato dextrose agar
pmol: Parts per million
RACE: Rapid amplification of cDNA ends
RdRP: RNA-dependent RNA polymerase
Rpm: Revolutions per minute
rRNA: Ribosomal RNA (rRNA);
RT-PCR: Reverse-transcription polymerase chain reaction
ssDNA: Single-strand DNA
ssRNA: Single-strand RNA
T525: The 525 strain with mycovirus
T525-F: The 525-F strain without mycovirus
TDT: Terminal deoxynucleotidyl transferase
UV: Ultraviolet
μl: Microliter
μM: 1 pmol/μL
