# Supplementary material for: A novel double-stranded RNA mycovirus isolated from Trichoderma harzianum
Source: Virol J. 2019 Sep 11;16:113. doi: 10.1186/s12985-019-1213-x (PMC6737671; doi:10.1186/s12985-019-1213-x)
Supplement: Supplementary file 1 — Table S1. Information on Trichoderma strains collected from Xinjiang, Inner Mongolia, Jilin and Heilongjiang Provinces of China. Table S2. High identities between contig 36 and other mycoviruses as determined by next-generation sequencing (HGS). Table S3. The primers used for 5’ RACE and 3’ RACE of the mycovirus genome sequence. Table S4. RdRP data of mycoviruses used in the phylogenetic analysis, including the identities of RdRP between ThMV1 and the compared mycoviruses. Table S5. CP data of mycoviruses used in the phylogentic analysis, including the CP identities between ThMV1 and the compared mycoviruses. Table S6. RdRP+CP data of mycoviruses used in the phylogenetic analysis, including the identities of RdRP+ CP between ThMV1 and the compared mycoviruses. Table S7. Statistical analysis of hyphae from T525 and T525-F after 5 days on PDA medium by the Mann-Whitney U test. Table S8. Average growth rate, standard deviation, and statistical analysis of hyphae from T525 and T525-F after 8 days on CZA medium by the Mann-Whitney U test. Table S9. Biomass comparison, standard deviation, and statistical analysis of biomass between T525 and T525-F by the Mann-Whitney U test. Table S10. Statistical analysis for the comparison of biomass between T525 and T525-F by using SPSS. Figure S1. Electrophoresis images of 5’ RACE and 3’ RACE results for the genome sequence of the T525 mycovirus. Figure S2. The secondary RNA structures of the 5’UTR and 3’UTR. Figure S3. The antagonistic characteristics of T525 and T525-F against F. oxysporum f.sp. cucumebrium Owen, B. cinerea and F. oxysporum f. sp. vasinfectum. Figure S4. Experimental flow chart for evaluating the biocontrol capabilities of F. oxysporum f. sp. cucumebrium Owen in cucumber. (DOCX 293 kb) [file 12985_2019_1213_MOESM1_ESM.docx]

**Additional files:**

Supplemental Table 1. Information on *Trichoderma* spp. strains collected from Xinjiang, Inner Mongolia, Jilin and Heilongjiang Provinces of China

| The order of the isolates | Strain Number | Strain Name | Region |
| --- | --- | --- | --- |
| 1 | CTCCSJ-G-QT40002 | *Hypocrea lixii/Trichoderma harzianum* | Xinjiang |
| 2 | CTCCSJ-G-QT40003 | *Hypocrea lixii/Trichoderma harzianum* | Xinjiang |
| 3 | CTCCSJ-G-QT40004 | *Hypocrea lixii/Trichoderma harzianum* | Xinjiang |
| 4 | CTCCSJ-G-HB40005 | *Trichoderma viridescens* | Xinjiang |
| 5 | CTCCSJ-F-ZY40007 | *Hypocrea schweinitzii/Trichoderma citrinoviride* | Xinjiang |
| 6 | CTCCSJ-F-ZY40008 | *Hypocrea schweinitzii/Trichoderma citrinoviride* | Xinjiang |
| 7 | CTCCSJ-F-ZY40009 | *Hypocrea schweinitzii/Trichoderma citrinoviride* | Xinjiang |
| 8 | CTCCSJ-G-JK40012 | *Hypocrea lixii/Trichoderma harzianum* | Xinjiang |
| 9 | CTCCSJ-G-HB40015 | *Hypocrea lixii/Trichoderma harzianum* | Xinjiang |
| 10 | CTCCSJ-G-HB40017 | *Trichoderma harzianum* | Xinjiang |
| 11 | CTCCSJ-G-HB40019 | *Trichoderma afroharzianum* | Xinjiang |
| 12 | CTCCSJ-F-ZY40020 | *Hypocrea schweinitzii/Trichoderma citrinoviride* | Xinjiang |
| 13 | CTCCSJ-F-KY40021 | *Hypocrea schweinitzii/Trichoderma citrinoviride* | Xinjiang |
| 14 | CTCCSJ-F-ZY40022 | *Hypocrea schweinitzii/Trichoderma citrinoviride* | Xinjiang |
| 15 | CTCCSJ-F-ZY40023 | *Hypocrea schweinitzii/Trichoderma citrinoviride* | Xinjiang |
| 16 | CTCCSJ-G-HB40030 | *Hypocrea pseudoharzianum* | Inner Mongolia |
| 17 | CTCCSJ-G-HB40031 | *Hypocrea pseudoharzianum* | Inner Mongolia |
| 18 | CTCCSJ-G-HB40032 | *Hypocrea schweinitzii/Trichoderma citrinoviride* | Inner Mongolia |
| 19 | CTCCSJ-G-HB40038 | *Hypocrea schweinitzii/Trichoderma citrinoviride* | Inner Mongolia |
| 20 | CTCCSJ-G-HB40040 | *Trichoderma longibrachiatum* | Inner Mongolia |
| 21 | CTCCSJ-G-HB40046 | *Hypocrea schweinitzii/Trichoderma citrinoviride* | Inner Mongolia |
| 22 | CTCCSJ-F-ZY40048 | *Trichoderma afroharzianum* | Xinjiang |
| 23 | CTCCSJ-G-HB40050 | *Trichoderma afroharzianum* | Inner Mongolia |
| 24 | CTCCSJ-F-KY40053 | *Trichoderma afroharzianum* | Inner Mongolia |
| 25 | CTCCSJ-G-HB40055 | *Trichoderma afroharzianum* | Xinjiang |
| 26 | CTCCSJ-G-HB40057 | *Trichoderma longibrachiatum* | Inner Mongolia |
| 27 | CTCCSJ-G-HB40061 | *Trichoderma afroharzianum* | Inner Mongolia |
| 28 | CTCCSJ-G-HB40065 | *Trichoderma afroharzianum* | Inner Mongolia |
| 29 | CTCCSJ-G-JK40067 | *Trichoderma afroharzianum* | Xinjiang |
| 30 | CTCCSJ-G-HB40091 | *Hypocrea pseudoharzianum* | Inner Mongolia |
| 31 | CTCCSJ-G-QT40119 | *Trichoderma viridescens* | Jilin |
| 32 | CTCCSJ-F-ZY40121 | *Trichoderma koningiopsis* | Jilin |
| 33 | CTCCSJ-G-HB40137 | *Trichoderma harzianum* | Xinjiang |
| 34 | CTCCSJ-G-HB40142 | *Trichoderma afroharzianum* | Xinjiang |
| 35 | CTCCSJ-G-QT40148 | *Trichoderma longibrachiatum* | Inner Mongolia |
| 36 | CTCCSJ-G-QT40151 | *Trichoderma longibrachiatum* | Inner Mongolia |
| 37 | CTCCSJ-F-KY40153 | *Trichoderma longibrachiatum* | Inner Mongolia |
| 38 | CTCCSJ-G-QT40154 | *Trichoderma longibrachiatum* | Inner Mongolia |
| 39 | CTCCSJ-F-KY40155 | *Trichoderma longibrachiatum* | Inner Mongolia |
| 40 | CTCCSJ-G-HB40167 | *Trichoderma longibrachiatum* | Inner Mongolia |
| 41 | CTCCSJ-G-QT40168 | *Trichoderma longibrachiatum* | Inner Mongolia |
| 42 | CTCCSJ-G-HB40188 | *Trichoderma longibrachiatum* | Xinjiang |
| 43 | CTCCSJ-G-HB40189 | *Trichoderma longibrachiatum* | Xinjiang |
| 44 | CTCCSJ-G-HB40190 | *Trichoderma longibrachiatum* | Xinjiang |
| 45 | CTCCSJ-G-QT40192 | *Trichoderma longibrachiatum* | Xinjiang |
| 46 | CTCCSJ-G-QT40194 | *Trichoderma longibrachiatum* | Inner Mongolia |
| 47 | CTCCSJ-G-HB40218 | *Trichoderma longibrachiatum* | Inner Mongolia |
| 48 | CTCCSJ-G-QT40222 | *Trichoderma longibrachiatum* | Inner Mongolia |
| 49 | CTCCSJ-G-QT40223 | *Trichoderma longibrachiatum* | Inner Mongolia |
| 50 | CTCCSJ-G-HB40226 | *Trichoderma longibrachiatum* | Xinjiang |
| 51 | CTCCSJ-G-JK40241 | *Trichoderma longibrachiatum* | Xinjiang |
| 52 | CTCCSJ-G-JK40242 | *Trichoderma longibrachiatum* | Xinjiang |
| 53 | CTCCSJ-G-HB40244 | *Trichoderma afroharzianum* | Inner Mongolia |
| 54 | CTCCSJ-F-ZY40245 | *Trichoderma afroharzianum* | Inner Mongolia |
| 55 | CTCCSJ-F-ZY40246 | *Trichoderma afroharzianum* | Xinjiang |
| 56 | CTCCSJ-F-ZY40248 | *Trichoderma longibrachiatum* | Xinjiang |
| 57 | CTCCSJ-G-QT40252 | *Trichoderma afroharzianum* | Inner Mongolia |
| 58 | CTCCSJ-G-JK40253 | *Trichoderma afroharzianum* | Xinjiang |
| 59 | CTCCSJ-G-HB40257 | *Trichoderma longibrachiatum* | Inner Mongolia |
| 60 | CTCCSJ-G-HB40263 | *Trichoderma longibrachiatum* | Inner Mongolia |
| 61 | CTCCSJ-F-ZY40265 | *Trichoderma longibrachiatum* | Xinjiang |
| 62 | CTCCSJ-G-HB40270 | *Trichoderma longibrachiatum* | Xinjiang |
| 63 | CTCCSJ-G-QT40271 | *Trichoderma longibrachiatum* | Inner Mongolia |
| 64 | CTCCSJ-G-QT40274 | *Trichoderma longibrachiatum* | Inner Mongolia |
| 65 | CTCCSJ-G-HB40275 | *Trichoderma longibrachiatum* | Inner Mongolia |
| 66 | CTCCSJ-G-HB40276 | *Trichoderma longibrachiatum* | Xinjiang |
| 67 | CTCCSJ-F-KY40280 | *Trichoderma longibrachiatum* | Inner Mongolia |
| 68 | CTCCSJ-G-QT40283 | *Trichoderma longibrachiatum* | Inner Mongolia |
| 69 | CTCCSJ-G-HB40284 | *Trichoderma afroharzianum* | Inner Mongolia |
| 70 | CTCCSJ-G-JK40288 | *Trichoderma afroharzianum* | Xinjiang |
| 71 | CTCCSJ-G-QT40290 | *Trichoderma afroharzianum* | Inner Mongolia |
| 72 | CTCCSJ-G-JK40292 | *Trichoderma afroharzianum* | Xinjiang |
| 73 | CTCCSJ-F-ZY40300 | *Trichoderma afroharzianum* | Inner Mongolia |
| 74 | CTCCSJ-G-DK40302 | *Trichoderma longibrachiatum* | Inner Mongolia |
| 75 | CTCCSJ-G-QT40303 | *Trichoderma asperelloides* | Xinjiang |
| 76 | CTCCSJ-G-QT40304 | *Trichoderma asperelloides* | Inner Mongolia |
| 77 | CTCCSJ-G-HB40311 | *Trichoderma viridescens* | Heilongjiang |
| 78 | CTCCSJ-G-QT40314 | *Trichoderma koningiopsis* | Inner Mongolia |
| 79 | CTCCSJ-G-QT40315 | *Trichoderma koningiopsis* | Inner Mongolia |
| 80 | CTCCSJ-G-HB40322 | *Trichoderma viridescens* | Xinjiang |
| 81 | CTCCSJ-G-QT40323 | *Trichoderma viridescens* | Jilin |
| 82 | CTCCSJ-G-QT40324 | *Trichoderma koningiopsis* | Inner Mongolia |
| 83 | CTCCSJ-G-HB40325 | *Trichoderma koningiopsis* | Inner Mongolia |
| 84 | CTCCSJ-G-HB40327 | *Trichoderma koningiopsis* | Inner Mongolia |
| 85 | CTCCSJ-G-HB40340 | *Hypocrea lixii/Trichoderma harzianum* | Inner Mongolia |
| 86 | CTCCSJ-F-ZY40342 | *Trichoderma afroharzianum* | Xinjiang |
| 87 | CTCCSJ-F-ZY40354 | *Trichoderma afroharzianum* | Xinjiang |
| 88 | CTCCSJ-G-QT40356 | *Trichoderma afroharzianum* | Inner Mongolia |
| 89 | CTCCSJ-F-ZY40358 | *Trichoderma longibrachiatum* | Jilin |
| 90 | CTCCSJ-G-HB40372 | *Trichoderma longibrachiatum* | Xinjiang |
| 91 | CTCCSJ-G-HB40373 | *Trichoderma longibrachiatum* | Inner Mongolia |
| 92 | CTCCSJ-G-JK40376 | *Hypocrea lixii/Trichoderma harzianum* | Xinjiang |
| 93 | CTCCSJ-G-QT40408 | *Trichoderma longibrachiatum* | Inner Mongolia |
| 94 | CTCCSJ-G-HB40421 | *Hypocrea atroviridis* | Xinjiang |
| 95 | CTCCSJ-G-HB40423 | *Hypocrea koningii/Trichoderma koningii* | Xinjiang |
| 96 | CTCCSJ-G-HB40425 | *Hypocrea atroviridis* | Xinjiang |
| 97 | CTCCSJ-G-HB40429 | *Trichoderma longibrachiatum* | Xinjiang |
| 98 | CTCCSJ-G-HB40430 | *Trichoderma viridescens* | Xinjiang |
| 99 | CTCCSJ-F-ZYB40431 | *Trichoderma viridescens* | Xinjiang |
| 100 | CTCCSJ-G-HB40433 | *Hypocrea lixii/Trichoderma harzianum* | Xinjiang |
| 101 | CTCCSJ-G-QT40435 | *Hypocrea lixii/Trichoderma harzianum* | Xinjiang |
| 102 | CTCCSJ-G-HB40436 | *Hypocrea atroviridis* | Xinjiang |
| 103 | CTCCSJ-F-ZY40439 | *Trichoderma viridescens* | Xinjiang |
| 104 | CTCCSJ-G-HB40440 | *Trichoderma viridescens* | Xinjiang |
| 105 | CTCCSJ-G-HB40441 | *Hypocrea lixii/Trichoderma harzianum* | Xinjiang |
| 106 | CTCCSJ-G-QT40442 | *Hypocrea lixii/Trichoderma harzianum* | Xinjiang |
| 107 | CTCCSJ-G-HB40444 | *Hypocrea lixii/Trichoderma harzianum* | Xinjiang |
| 108 | CTCCSJ-G-QT40447 | *Hypocrea lixii/Trichoderma harzianum* | Xinjiang |
| 109 | CTCCSJ-G-HB40448 | *Hypocrea koningii/Trichoderma koningii* | Xinjiang |
| 110 | CTCCSJ-F-ZY40453 | *Hypocrea koningii/Trichoderma koningii* | Xinjiang |
| 111 | CTCCSJ-G-HB40455 | *Hypocrea lixii/Trichoderma harzianum* | Xinjiang |
| 112 | CTCCSJ-G-HB40456 | *Trichoderma gamsii* | Xinjiang |
| 113 | CTCCSJ-G-HB40461 | *Trichoderma guizhouense* | Inner Mongolia |
| 114 | CTCCSJ-G-HB40462 | *Hypocrea lixii/Trichoderma harzianum* | Inner Mongolia |
| 115 | CTCCSJ-G-QT40476 | *Trichoderma koningiopsis* | Inner Mongolia |
| 116 | CTCCSJ-G-HB40478 | *Hypocrea lixii/Trichoderma harzianum* | Inner Mongolia |
| 117 | CTCCSJ-G-HB40481 | *Hypocrea lixii/Trichoderma harzianum* | Inner Mongolia |
| 118 | CTCCSJ-G-HB40482 | *Hypocrea lixii/Trichoderma harzianum* | Inner Mongolia |
| 119 | CTCCSJ-G-HB40483 | *Hypocrea lixii/Trichoderma harzianum* | Inner Mongolia |
| 120 | CTCCSJ-G-HB40484 | *Trichoderma rossicum* | Inner Mongolia |
| 121 | CTCCSJ-G-HB40485 | *Trichoderma koningiopsis* | Inner Mongolia |
| 122 | CTCCSJ-G-QT40486 | *Hypocrea atroviridis* | Inner Mongolia |
| 123 | CTCCSJ-G-HB40487 | *Hypocrea lixii/Trichoderma harzianum* | Inner Mongolia |
| 124 | CTCCSJ-G-HB40490 | *Hypocrea lixii/Trichoderma harzianum* | Inner Mongolia |
| 125 | CTCCSJ-G-HB40495 | *Trichoderma koningiopsis* | Inner Mongolia |
| 126 | CTCCSJ-G-HB40496 | *Hypocrea schweinitzii/Trichoderma citrinoviride* | Inner Mongolia |
| 127 | PDA X35-1 | *Hypocrea schweinitzii/Trichoderma citrinoviride* | Inner Mongolia |
| 128 | XZ X46-1 | *Trichoderma koningiopsis* | Inner Mongolia |
| 129 | XZ N236-1 | *Trichoderma gamsii* | Inner Mongolia |
| 130 | PDA N239-3 | *Trichoderma hamatum* | Inner Mongolia |
| 131 | XZ N71-3 | *H.schweinitzii/T. citrinoviride* | Inner Mongolia |
| 132 | XZ X171-1 | *Hypocrea lixii/Trichoderma harzianum* | Inner Mongolia |
| 133 | PDA N182-1(525) | *Trichoderma harzianum* | Inner Mongolia |
| 134 | CTCCSJ-G-HB40547 | *Trichoderma harzianum* | Xinjiang |
| 135 | CTCCSJ-G-HB40551 | *Trichoderma harzianum* | Xinjiang |
| 136 | CTCCSJ-G-HB40565 | *Trichoderma harzianum* | Xinjiang |
| 137 | CTCCSJ-G-HB40582 | *Trichoderma harzianum* | Xinjiang |
| 138 | CTCCSJ-G-HB40609 | *Trichoderma harzianum* | Xinjiang |
| 139 | CTCCSJ-G-HB40613 | *Trichoderma harzianum* | Xinjiang |
| 140 | CTCCSJ-G-HB40614 | *Trichoderma harzianum* | Xinjiang |
| 141 | CTCCSJ-G-HB40615 | *Trichoderma harzianum* | Xinjiang |
| 142 | CTCCSJ-G-HB40616 | *Trichoderma harzianum* | Xinjiang |
| 143 | CTCCSJ-G-HB40618 | *Trichoderma harzianum* | Xinjiang |
| 144 | CTCCSJ-G-HB40672 | *Trichoderma harzianum* | Xinjiang |
| 145 | CTCCSJ-G-HB40732 | *Trichoderma harzianum* | Xinjiang |
| 146 | CTCCSJ-G-HB40733 | *Trichoderma harzianum* | Xinjiang |
| 147 | CTCCSJ-F-KZ40809 | *Trichoderma harzianum* | Xinjiang |
| 148 | CTCCSJ-G-JK40974 | *Trichoderma harzianum* | Inner Mongolia |
| 149 | CTCCSJ-G-HB40989 | *Trichoderma harzianum* | Inner Mongolia |
| 150 | CTCCSJ-G-QT40994 | *Trichoderma atroviride* | Heilongjiang |

| **Contig** | **Name of mycovirus** | **Accession Number** | **Identity** |
| --- | --- | --- | --- |
| contig36 | Alternaria longipes dsRNA virus 1 | YP_009052469.1 | 63.50% |
|  | Penicillium janczewskii Beauveria bassiana-like virus 1 | ALO50135.1 | 57.30% |
|  | Beauveria bassiana RNA virus 1 | AKC57301.1 | 57% |

Supplemental table 2. High identities between contig 36 and other mycoviruses as determined by next-generation sequencing (HGS)

Supplemental table 3. The primers used for 5’ RACE and 3’ RACE of the mycovirus genome sequence

| **Primer name** | **Primer sequence (from 5’ to 3’)** | **RACE step where used** |
| --- | --- | --- |
| 5’R1 | ACGAGAGCTTGACATTCCTTGG | 5’RACE (Reverse transcription) |
| 5’R2 | TCGCAAGGAATGGCTGTCTCG | 5’RACE (1st PCR) |
| 5’R3 | AACTTCTTCCCGCCTGCCTTG | 5’RACE (2nd PCR) |
| 3’R1 | ACTGGCTTCCTCCTTGACTGAGTC | 3’RACE (1st PCR) |
| 3’R2 | ACAGCTGCGACTCCACTATCACC | 3’RACE (2nd PCR) |

Supplemental Table 4. RdRP data of mycoviruses used in the phylogenetic analysis, including the identities of RdRP between ThMV1 and the compared mycoviruses.

| **Accession Number of RdRP** | **Mycovirus name** | **Classification** | **The host** | **Identity of the whole protein sequence (coverage) according to the EMBOSS needle program** |
| --- | --- | --- | --- | --- |
| CEZ26307.1 | Sclerotinia sclerotiorum dsRNA mycovirus-L | unclassified *Fusagraviridae* | *Sclerotinia sclerotiorum* | 9.40% |
| YP 006331065.1 | Sclerotinia sclerotiorum dsRNA mycovirus-L | unclassified *Fusagraviridae* | *Sclerotinia sclerotiorum* | 7.90% |
| CEZ26308.1 | Sclerotinia sclerotiorum dsRNA mycovirus-L | unclassified *Fusagraviridae* | *Sclerotinia sclerotiorum* | 9.50% |
| YP 009115498.1 | Botrytis cinerea RNA virus 1 | unclassified *Fusagraviridae* | *Botrytis* cinerea | 10.10% |
| YP 003288789.1 | Fusarium graminearum dsRNA mycovirus-3 | unclassified *Fusagraviridae* | *Fusarium* *graminearum* | 10.20% |
| YP 009253997.1 | Fusarium poae dsRNA virus 3 | unclassified *Fusagraviridae* | *Fusarium* *poae* | 7.30% |
| ALD89097.1 | Macrophomina phaseolina double-stranded RNA virus 2 | unclassified *Fusagraviridae* | *Macrophomina phaseolina* | 9.90% |
| AEZ54146.1 | Fusarium virguliforme dsRNA mycovirus 2 | unclassified *Fusagraviridae* | *Fusarium virguliforme* | 8.80% |
| AEZ54148.1 | Fusarium virguliforme dsRNA mycovirus 1 | unclassified *Fusagraviridae* | *Fusarium virguliforme* | 9.50% |
| CAJ34335.2 | Phlebiopsis gigantea mycovirus dsRNA 2 | unclassified *Fusagraviridae* | *Phlebiopsis gigantea* | 11.90% |
| YP 003359178.1 | Diplodia scrobiculata RNA virus 1 | unclassified *Fusagraviridae* | *Diplodia scrobiculata* | 12.80% |
| AEX87902.1 | Phytophthora infestans RNA virus 3 | unclassified *Fusagraviridae* | *Phytophthora infestans* | 12.80% |
| YP 003288763.1 | Rosellinia necatrix megabirnavirus 1/W779 | *Megabirnaviridae*  *Megabirnavirus* | *Rosellinia necatrix* | 10.30% |
| YP 003541123.1 | Phlebiopsis gigantea mycovirus dsRNA 1 | *Unclassified*  *Totiviridae* | *Phlebiopsis gigantea* | 9.50% |
| YP 392482.1 | Penicillium chrysogenum virus | *Chrysoviridae*  *Chrysovirus* | *Penicillium chrysogenum* | 6.30% |
| ABQ53134.1 | Fusarium oxysporum chrysovirus 1 | *Chrysoviridae*  *Chrysovirus* | *Fusarium oxysporum* | 4.50% |
| YP 052858.1 | Helminthosporium victoriae 145S virus | *Chrysoviridae*  *Chrysovirus* | *Helminthosporium victoriae* | 10.00% |
| NP 041191.1 | Leishmania RNA virus 1 - 1 | *Totiviridae*  *Leishmaniavirus* | *Leishmania* | 13.60% |
| NP 043465.1 | Leishmania RNA virus 2 - 1 | *Totiviridae*  *Leishmaniavirus* | *Leishmania* | 13.40% |
| NP 047560.1 | Sphaeropsis sapinea RNA virus 2 | *Totiviridae*  *Victorivirus* | *Sphaeropsis sapinea* | 13.00% |
| AAB94791.2 | Helminthosporium victoriae virus 190S | *Totiviridae*  *Victorivirus* | *Helminthosporium victoriae* | 7.40% |
| NP 047558.1 | Sphaeropsis sapinea RNA virus 1 | *Totiviridae*  *Victorivirus* | *Sphaeropsis sapinea* | 9.60% |
| YP 122352.1 | Magnaporthe oryzae virus 1 | *Totiviridae*  *Victorivirus* | *Magnaporthe oryzae* | 11.20% |
| YP 009052469.1 | Alternaria longipes dsRNA virus 1 | unclassified mycoviruses | *Alternaria longipes* | 62.20% |
| YP 009177217.1 | Colletotrichum higginsianum non-segmented dsRNA virus 1 | unclassified mycoviruses | *Colletotrichum higginsianum* | 52.40% |
| ALO50135.1 | Penicillium janczewskii Beauveria bassiana-like virus 1 | unclassified mycoviruses | *Penicillium janczewskii Beauveria bassiana* | 56.60% |
| AKC57301.1 | Beauveria bassiana RNA virus 1 | unclassified mycoviruses | *Beauveria bassiana* | 56.00% |
| YP 009154711.1 | Beauveria bassiana RNA virus 1 | unclassified mycoviruses | *Beauveria bassiana* | 56.10% |

Supplemental Table 5. CP data of mycoviruses used in the phylogentic analysis, including the CP identities between ThMV1 and the compared mycoviruses.

| **Accession Number of Hypothetical protein or CP** | **Mycovirus Name** | **Classification** | **The host** | **Identity of the whole protein sequence (coverage) according to the EMBOSS needle program** |
| --- | --- | --- | --- | --- |
| YP_006331064.1 | Sclerotinia sclerotiorum dsRNA mycovirus-L | Unclassified  *Fusagraviridae* | *Sclerotinia sclerotiorum* | 6.00% |
| YP_009115497.1 | Botrytis cinerea RNA virus 1 | Unclassified  *Fusagraviridae* | *Botrytis cinerea* | 5.30% |
| YP_009253996.1 | Fusarium poae dsRNA virus 3 | Unclassified  *Fusagraviridae* | *Fusarium poae* | 5.70% |
| YP_003288788.1 | Fusarium graminearum dsRNA mycovirus-3 | Unclassified  *Fusagraviridae* | *Fusarium graminearum* | 4.80% |
| ALD89096.1 | Macrophomina phaseolina double-stranded RNA virus 2 | Unclassified  *Fusagraviridae* | *Macrophomina phaseolina* | 5.70% |
| AEZ54147.1 | Fusarium virguliforme dsRNA mycovirus 1 | Unclassified  *Fusagraviridae* | *Fusarium virguliforme* | 3.80% |
| AEZ54145.1 | Fusarium virguliforme dsRNA mycovirus 2 | Unclassified  *Fusagraviridae* | *Fusarium virguliforme* | 7.10% |
| YP_392484.1 | Penicillium chrysogenum virus | *Chrysoviridae*  *Chrysovirus* | *Penicillium chrysogenum* | 6.10% |
| YP_003359177.1 | Diplodia scrobiculata RNA virus 1 | Unclassified  *Fusagraviridae* | *Diplodia scrobiculata* | 5.60% |
| AEX87901.1 | Phytophthora infestans RNA virus 3 | Unclassified  *Fusagraviridae* | *Phytophthora infestans* | 6.90% |
| YP_003288762.1 | Rosellinia necatrix megabirnavirus 1/W779 | *Megabirnaviridae*  *Megabirnavirus* | *Rosellinia necatrix* | 8.50% |
| NP_041190.1 | Leishmania RNA virus 1 - 1 | *Totiviridae*  *Leishmaniavirus* | *Leishmania* | 10.60% |
| NP_043464.1 | Leishmania RNA virus 2 - 1 | *Totiviridae*  *Leishmaniavirus* | *Leishmania* | 3.60% |
| NP_047559.1 | Sphaeropsis sapinea RNA virus 2 | *Totiviridae*  *Leishmaniavirus* | *Sphaeropsis sapinea* | 11.40% |
| AAB94790.2 | Helminthosporium victoriae virus 190S | *Totiviridae*  *Leishmaniavirus* | *Helminthosporium victoriae* | 4.40% |
| YP_122351.1 | Magnaporthe oryzae virus 1 | *Totiviridae*  *Leishmaniavirus* | *Magnaporthe oryzae* | 4.40% |
| NP_047557.1 | Sphaeropsis sapinea RNA virus 1 | *Totiviridae*  *Leishmaniavirus* | *Sphaeropsis sapinea* | 3.50% |
| NP_620494.1 | Saccharomyces cerevisiae virus L-A | *Totiviridae*  *Totivirus* | *Saccharomyces cerevisiae* | 1.90% |
| YP_009052468.1 | Alternaria longipes dsRNA virus 1 | unclassified mycoviruses | *Alternaria longipes* | 34.50% |
| YP_009177216.1 | Colletotrichum higginsianum  non-segmented dsRNA virus 1 | unclassified mycoviruses | *Colletotrichum higginsianum* | 28.50% |
| YP_009154710.1 | Beauveria bassiana RNA virus 1 | unclassified mycoviruses | *Beauveria bassiana* | 30.20% |
| AKC57300.1 | Beauveria bassiana RNA virus 1 | unclassified mycoviruses | *Beauveria bassiana* | 29.70% |

Supplemental Table 6. RdRP+CP data of mycoviruses used in the phylogenetic analysis, including the identities of RdRP+ CP between ThMV1 and the compared mycoviruses.

| **Mycovirus name** | **Classification** | **The host** | **Identity of the whole protein sequence (coverage) according to the EMBOSS needle program** |
| --- | --- | --- | --- |
| Sclerotinia sclerotiorum dsRNA mycovirus-L | unclassified *Fusagraviridae* | *Sclerotinia sclerotiorum* | 6.80% |
| Sclerotinia sclerotiorum dsRNA mycovirus-L | unclassified *Fusagraviridae* | *Sclerotinia sclerotiorum* | 7.80% |
| Botrytis cinerea RNA virus 1 | unclassified *Fusagraviridae* | *Botrytis* cinerea | 7.90% |
| Fusarium graminearum dsRNA mycovirus-3 | unclassified *Fusagraviridae* | *Fusarium* *graminearum* | 7.90% |
| Fusarium poae dsRNA virus 3 | unclassified *Fusagraviridae* | *Fusarium* *poae* | 7.10% |
| Macrophomina phaseolina double-stranded RNA virus 2 | unclassified *Fusagraviridae* | *Macrophomina phaseolina* | 8.00% |
| Fusarium virguliforme dsRNA mycovirus 2 | unclassified *Fusagraviridae* | *Fusarium virguliforme* | 9.20% |
| Fusarium virguliforme dsRNA mycovirus 1 | unclassified *Fusagraviridae* | *Fusarium virguliforme* | 6.80% |
| Diplodia scrobiculata RNA virus 1 | unclassified *Fusagraviridae* | *Diplodia scrobiculata* | 15.00% |
| Phytophthora infestans RNA virus 3 | unclassified *Fusagraviridae* | *Phytophthora infestans* | 8.80% |
| Rosellinia necatrix megabirnavirus 1/W779 | *Megabirnaviridae*  *Megabirnavirus* | *Rosellinia necatrix* | 8.60% |
| Penicillium chrysogenum virus | *Chrysoviridae*  *Chrysovirus* | *Penicillium chrysogenum* | 9.30% |
| Leishmania RNA virus 1 - 1 | *Totiviridae*  *Leishmaniavirus* | *Leishmania* | 1.90% |
| Leishmania RNA virus 2 - 1 | *Totiviridae*  *Leishmaniavirus* | *Leishmania* | 8.70% |
| Sphaeropsis sapinea RNA virus 2 | *Totiviridae*  *Victorivirus* | *Sphaeropsis sapinea* | 13.00% |
| Helminthosporium victoriae virus 190S | *Totiviridae*  *Victorivirus* | *Helminthosporium victoriae* | 9.20% |
| Sphaeropsis sapinea RNA virus 1 | *Totiviridae*  *Victorivirus* | *Sphaeropsis sapinea* | 8.00% |
| Magnaporthe oryzae virus 1 | *Totiviridae*  *Victorivirus* | *Magnaporthe oryzae* | 8.70% |
| Alternaria longipes dsRNA virus 1 | unclassified mycoviruses | *Alternaria longipes* | 50.50% |
| Colletotrichum higginsianum non-segmented dsRNA virus 1 | unclassified mycoviruses | *Colletotrichum higginsianum* | 44.00% |
| Beauveria bassiana RNA virus 1 | unclassified mycoviruses | *Beauveria bassiana* | 46.40% |
| Beauveria bassiana RNA virus 1 | unclassified mycoviruses | *Beauveria bassiana* | 46.60% |

Supplemental Table 7. Statistical analysis of hyphae from T525 and T525-F after 5 days on PDA medium by the Mann-Whitney U test.

| **Replicate** | **Strain 525(mm/d)** | **Strain 525-F(mm/d)** |
| --- | --- | --- |
| 1 | 21.50667 | 20.70667 |
| 2 | 20.14000 | 20.42333 |
| 3 | 21.90333 | 20.8000 |
| 4 | 20.47667 | 20.63667 |
| 5 | 21.01333 | 20.51667 |
| 6 | 21.31333 | 20.35333 |
| 7 | 20.77333 | 20.89667 |
| 8 | 21.96333 | 20.57667 |
| 9 | 20.67000 | 20.68000 |
| 10 | 20.72333 | 21.86667 |
| 11 | 20.48333 | 20.60667 |
| 12 | 20.03000 | 21.01333 |
| 13 | 20.98667 | 20.66333 |
| 14 | 20.70667 | 20.35000 |
| 15 | 20.47000 | 21.61000 |
| 16 | 21.11333 | 21.29667 |
| 17 | 21.31333 | 18.93000 |
| 18 | 20.66000 | 21.51000 |
| 19 | 20.65667 | 20.98333 |
| 20 | 21.24333 | 21.20667 |
| 21 | 20.97667 | 20.99333 |
| 22 | 20.48000 | 20.90000 |
| 23 | 20.80333 | 20.66667 |
| 24 | 20.50333 | 20.37000 |
| 25 | 21.43667 | 20.89667 |
| 26 | 21.15667 | 20.39333 |
| 27 | 21.06000 | 20.30333 |
| 28 | 20.68667 | 19.92333 |
| 29 | 21.29333 | 24.01667 |
| 30 | 20.43333 | 20.19667 |
| Standard deviations | 0.458397113407411 | 0.800489541576178 |
| Statistical significance | 0.602645^#^ | |

^#^: Statistical difference between two strains

Supplemental table 8. Average growth rate, standard deviation, and statistical analysis of hyphae from T525 and T525-F after 8 days on CZA medium by the Mann-Whitney U test.

| **Repeats** | **Strain 525(mm/d)** | **Strain 525-F(mm/d)** |
| --- | --- | --- |
| 1 | 10.28571 | 9.018571 |
| 2 | 8.90000 | 9.980000 |
| 3 | 9.260000 | 8.557143 |
| 4 | 10.42714 | 8.881429 |
| 5 | 10.43571 | 9.121429 |
| 6 | 9.332857 | 9.310000 |
| 7 | 10.31286 | 8.995714 |
| 8 | 10.40571 | 9.047143 |
| 9 | 10.28571 | 8.911429 |
| 10 | 10.00286 | 8.858571 |
| 11 | 8.985714 | 9.000000 |
| 12 | 9.101429 | 8.897143 |
| 13 | 9.027143 | 9.862857 |
| 14 | 9.664286 | 8.657143 |
| 15 | 8.980000 | 8.891429 |
| 16 | 9.168571 | 10.00571 |
| 17 | 9.054286 | 9.351429 |
| 18 | 10.07571 | 9.502857 |
| 19 | 8.777143 | 9.322857 |
| 20 | 9.088571 | 9.080000 |
| 21 | 9.228571 | 8.675714 |
| 22 | 8.652857 | 9.917143 |
| 23 | 9.107143 | 9.055714 |
| 24 | 9.372857 | 9.570000 |
| 25 | 9.542857 | 9.048571 |
| 26 | 9.078571 | 8.552857 |
| 27 | 8.942857 | 9.108571 |
| 28 | 8.630000 | 9.567143 |
| 29 | 10.11571 | 10.00286 |
| 30 | 9.204286 | 8.328571 |
| Standard deviations | 0.577931178179563 | 0.450967174293484 |
| Statistical significance | 0.045008^#^ | |

^#^: Significant difference between the two strains

Supplemental table 9. Biomass comparison, standard deviation, and statistical analysis of biomass between T525 and T525-F by the Mann-Whitney U test.

| **Repeats** | **Strain 525(g)** | **Strain 525-F(g)** |
| --- | --- | --- |
| 1 | 0.352 | 0.297 |
| 2 | 0.390 | 0.290 |
| 3 | 0.444 | 0.318 |
| 4 | 0.394 | 0.255 |
| 5 | 0.420 | 0.323 |
| 6 | 0.570 | 0.338 |
| 7 | 0.419 | 0.205 |
| 8 | 0.430 | 0.259 |
| 9 | 0.416 | 0.359 |
| 10 | 0.280 | 0.453 |
| 11 | 0.383 | 0.323 |
| 12 | 0.375 | 0.388 |
| 13 | 0.279 | 0.418 |
| 14 | 0.401 | 0.422 |
| 15 | 0.380 | 0.396 |
| 16 | 0.360 | 0.423 |
| 17 | 0.477 | 0.374 |
| 18 | 0.506 | 0.403 |
| 19 | 0.272 | 0.368 |
| 20 | 0.386 | 0.393 |
| Average biomass | 0.3967 | 0.35025 |
| Standard deviations | 0.0709493481294929 | 0.0641520654383004 |
| Statistical significance | 0.04088683* | |

*: Significant difference between the two strains

Supplemental table 10. Statistical analysis for the comparison of biomass between T525 and T525-F by using SPSS.

| **Origin** | **Type III sum of squares** | **df** | **Mean square** | ***F*** | ***P*** |
| --- | --- | --- | --- | --- | --- |
| Corrected model | 4000.316^a^ | 3 | 1333.439 | 3713.903 | 0.000 |
| Intersection | 27294.512 | 1 | 27294.512 | 76020.877 | 0.000 |
| Strain | 1.019 | 1 | 1.019 | 2.838 | 0.095 |
| Media | 3999.029 | 1 | 3999.029 | 11138.125 | 0.000 |
| Strain by media | 0.269 | 1 | 0.269 | 0.748 | 0.389 |
| Error | 41.649 | 116 | 0.359 |  |  |
| Total | 31336.477 | 120 |  |  |  |
| Corrected Total | 4041.965 | 119 |  |  |  |

^a^ *R*^2^ = 0.990 (*R_adj_*^2^ = 0.989)


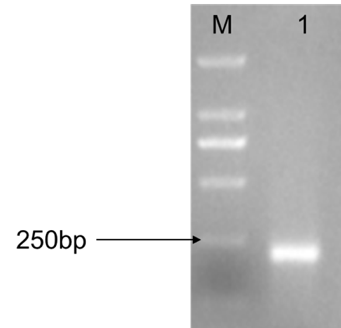

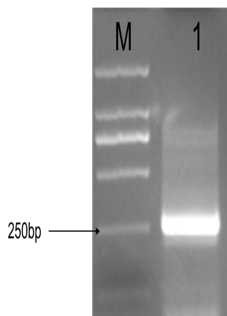


A. B.

Supplemental Figure 1. Electrophoresis images of 5’ RACE and 3’ RACE results for the genome sequence of the T525 mycovirus. A) Electrophoresis images of the 5’ RACE results; B) electrophoresis image of the 3’ RACE results.


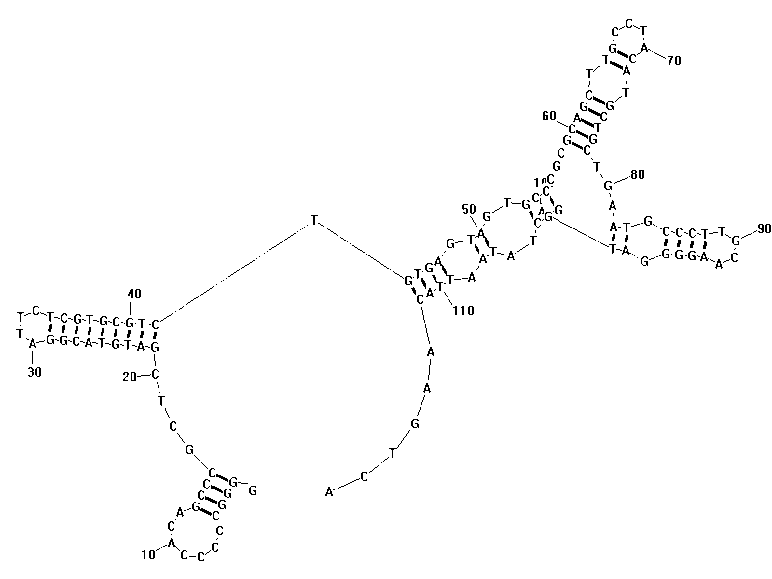


A.


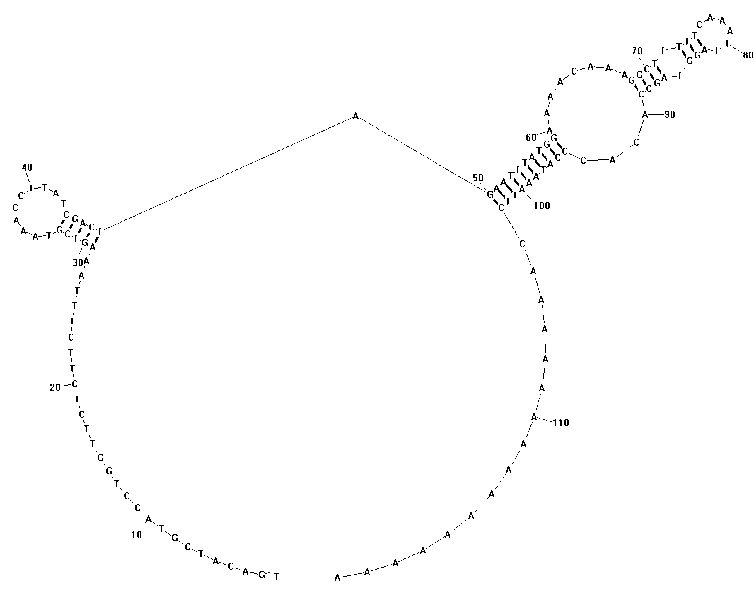


B.

Supplemental figure 2. The secondary RNA structures of the 5’UTR and 3’UTR. A: Secondary RNA structures of the 5’UTR, with an energy of -41.2 Kcal/mol; B: secondary RNA structures of the 3’UTR, with an energy of -18.7 Kcal/mol


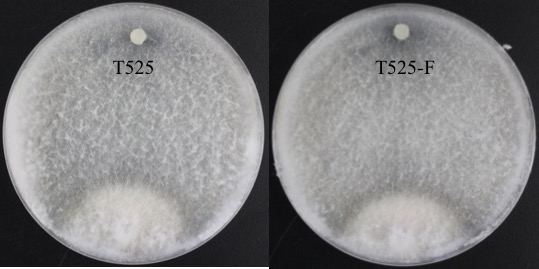

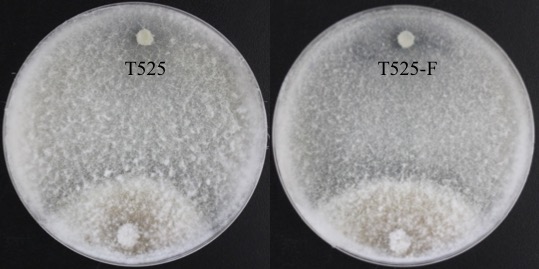


A. B.


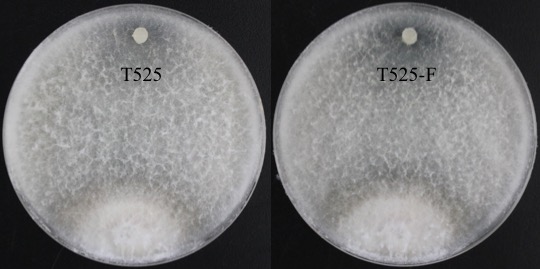


C.

### Supplemental figure 3. The antagonistic characteristics of T525 and T525-F against *F. oxysporum* f.sp. *cucumebrium* Owen, *B. cinerea* and [*F. oxysporum* f. sp. *vasinfectum*](http://www.baidu.com/link?url=GpBIC4JM3gi6gnCxy4r8fdusZMhFiGYigp3gEKuhkb6cpQ_2g_JbheHnXGz2EdAU). A: Antagonism of T525 and T525-F against *F. oxysporum* f.sp*. cucumebrium* Owen; B: antagonism of T525 and T525-F against *B. cinerea*; C: antagonism of T525 and T525-F against [*F. oxysporum* f. sp. *vasinfectum*](http://www.baidu.com/link?url=GpBIC4JM3gi6gnCxy4r8fdusZMhFiGYigp3gEKuhkb6cpQ_2g_JbheHnXGz2EdAU).

###
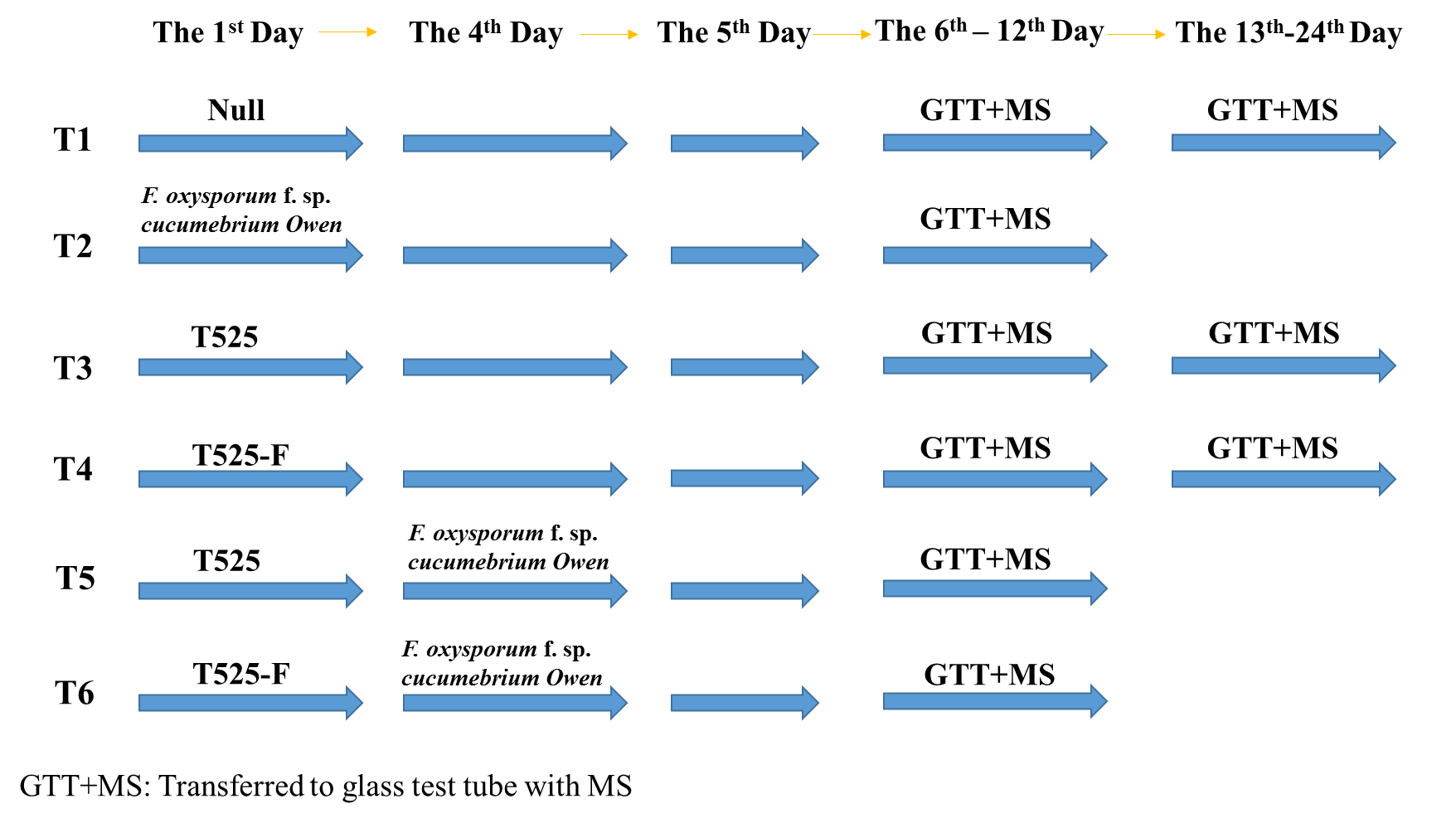


### Supplemental figure 4. Experimental flow chart for evaluating the biocontrol capabilities of *F. oxysporum f. sp. cucumebrium* Owen in cucumber.
